# Examining the impact of COVID‐19 on cardiac surgery services: The lessons learned from this pandemic

**DOI:** 10.1111/jocs.14783

**Published:** 2020-07-09

**Authors:** Amer Harky, Runzhi Chen, Mark Pullan

**Affiliations:** ^1^ University of Liverpool School of Medicine Liverpool UK; ^2^ Department of Cardiothoracic Surgery Liverpool Heart and Chest Liverpool UK; ^3^ Faculty of Medicine, Imperial College School of Medicine Imperial College London London UK

**Keywords:** cardiac surgery, COVID‐19, healthcare provisions, SARS‐CoV‐2

Under the unprecedented pressures of the global coronavirus disease 2019 (COVID‐19) pandemic, there is an urgent requisite for successful strategies to safely deliver cardiac surgery. Severe acute respiratory syndrome coronavirus 2 (SARS‐CoV‐2) was first described in early December 2019, and the rapid spread and emergence of this virus have caused significant disruptions in the delivery of healthcare services worldwide.^1^, ^2^ In particular, the provision of cardiac surgery has been disproportionally affected due to the reallocation of intensive care resources, such as ventilators.^2^ Additionally, patients with pre‐existing cardiovascular disease are likely to have comorbidities which are associated with poorer clinical outcomes in confirmed SARS‐CoV‐2 cases.^3^, ^4^ Despite this, Yandrapalli et al^5^ have reported the first case of a successful coronary artery bypass graft (CABG) operation in a patient with asymptomatic SARS‐CoV‐2 infection, which offers insights into how cardiac surgery could be adapted to solve the challenges of this pandemic.

In response to the burden of COVID‐19 on healthcare systems in the UK, elective cardiac surgeries have been delayed owing to the redistribution of intensive care resources and the unquantifiable risk of acquiring COVID‐19.^2^ Likewise, cardiac surgery services have undergone structural remodeling into a centralized system in an attempt to continue provisions of emergency surgery alongside hospital management of COVID‐19 patients.^2^ Unsurprisingly, most cardiac surgery units across the globe have seen a sharp decline in surgeries as a result, and one unit reported an 83% reduction in cardiac index cases between 23rd March to 4th May 2020.^2^ Similar models have been used in Europe to manage healthcare services and increase intensive care capacity. For example in the Lombardy region of Italy, 16 out of 20 cardiac surgical units discontinued services and all urgent cases have been consequently diverted to the remaining four units for centralized services.^6^ Although these measures have been beneficial for supporting the focused management of COVID‐19 patients, it is important to reflect upon the future consequences of delayed elective cardiac surgery. Indeed, such patients are likely to have progressive conditions and further work is needed to investigate the long‐term impact of COVID‐19 on mortality and morbidity in this cohort.

The case report by Yandrapalli et al^5^ highlights the importance of routine SARS‐CoV‐2 testing for all patients requiring cardiac surgery, especially for detecting asymptomatic or subclinical infections. Active SARS‐CoV‐2 infection may precipitate an overproduction of early response proinflammatory cytokines in postoperative period, leading to unfavorable surgical outcomes.^7^, ^8^ Moreover, preliminary studies have shown that patients with established cardiovascular diseases may have a greater risk of increased SARS‐CoV‐2 infection severity and prognosis.^9^ Taken together, assessment for active infection is crucial for risk stratification. In addition, clinicians should consider the threshold for surgery when selecting patients for cardiac surgery. An international, multicenter cohort study by COVIDSurg Collaborative which included 1128 confirmed SARS‐CoV‐2 patients undergoing a broad range of surgeries revealed that the 30‐day mortality risk was significantly associated with the patient demographics of male sex, an age of 70 years or older, and poor preoperative physical health status.^10^ Collectively, the risks and benefits of cardiac surgery should be carefully considered in such patients due to higher mortality risk.^10^ Alternative therapeutic procedures with rapid discharge, such as percutaneous intervention or medical therapy, may be more appropriate to reduce SARS‐CoV‐2 related mortality and nosocomial infection risk.^11^

Current evidence is limited for postoperative outcomes in cardiac surgery cases. In the aforementioned cohort study by COVIDSurg Collaborative, the 30‐day mortality rate was 23.8%.^10^ In addition, the study reported that 51.2% of patients had postoperative pulmonary complications, which was associated with a higher mortality rate of 38.0%.^10^ In another case report describing an emergency CABG operation, the asymptomatic patient succumbed to pulmonary complications arising from a SARS‐CoV‐2 infection confirmed postoperatively.^12^ The authors acknowledge that the undiagnosed infection may have triggered a refractory pathological response after cardiac surgery. Indeed, recent literature has suggested that patients with SARS‐CoV‐2 are at higher risk of developing thromboembolisms, possibly mediated by the interaction with angiotensin‐converting enzyme 2 (ACE2) receptors.^13^ Similarly, there is a consensus that SARS‐CoV‐2 has direct adverse effects on the myocardium due to high expression of ACE2.^14^ As such, SARS‐CoV‐2 can potentially trigger multisystem complications which require vigilant monitoring, especially in patients requiring cardiopulmonary bypass and at high risk of developing thromboembolisms. Cardiac surgery patients represent a vulnerable patient population, and this cohort may experience worse outcomes with SARS‐CoV‐2 infection based on the currently available evidence. In the latest recommendation, UK currently advises all patients who are listed for elective cardiac surgery to self‐isolate for 14 days before the surgery date, in a measure to limit and contain the exposure of such cohort to the smallest possibilities of acquiring COVID‐19.

Currently, the future of cardiac surgery after the pandemic is unclear as the evidence is still emerging. However, the lessons learned from these unprecedented times can be taken forward to inform future service planning. Moving forward, routine screening of patients for SARS‐CoV‐2 infection will undoubtedly play a key role in identifying asymptomatic or subclinical infections. The preoperative UK National Health Service testing recommendations should be broadened so that all patients undergoing cardiac surgery are screened, given the higher risk of postoperative complications in this population. Similarly, repeat testing is important for monitoring patients for concomitant infections. Alongside changes to hospital protocol, service delivery will inevitably shift. The successful application of telemedicine during the pandemic has already been reported in the delivery of oncology services.^15^ Moreover, the benefits of telecardiology outside of the COVID‐19 era have been previously reported, and cardiology services will likely embrace the utilization of telemedicine for managing outpatient consultations.^16^ Units will also have to address the vast backlog of surgeries caused by the cancellation of elective cardiac operations in a sustainable manner, with adequate hospital space and personal protective equipment availability.^17^ To resume success services, planning for this eventuality should begin now and patients at significant mortality risk due to delayed surgery need to be prioritized.

Ultimately, clear guidelines should be implemented to ensure the safe resumption of surgical services, while also reassuring patients concerned about safety.^3^ Although the future trajectory of this pandemic is uncertain, the insights from the impact of COVID‐19 on cardiac surgery will undoubtedly shape the future delivery of cardiac surgery.
